# Closing Editorial: From Emergency Care to Prevention and Innovation

**DOI:** 10.3390/epidemiologia7040118

**Published:** 2026-08-21

**Authors:** Giuseppe Stirparo, Giuseppe Ristagno

**Affiliations:** 1 SIMED—Società Italiana di Medicina e Divulgazione Scientifica, via Trento 41, 43122 Parma, Italy; 2 Fondazione IRCCS Ca’ Granda Ospedale Maggiore Policlinico, 20090 Milano, Italy

The completion of this Special Issue, “Acute Diseases and Epidemiological Investigations”, offers an opportunity to reflect on the breadth of research contributions received and on the evolving challenges that characterize modern public health and emergency medicine. As Guest Editors, we would like to express our sincere appreciation to all authors, reviewers, and editorial staff whose efforts contributed to the success of this collection.

This Special Issue was launched with the objective of gathering contemporary evidence on acute diseases and their epidemiological determinants, with a particular focus on emergency medical system organization, clinical outcomes, public health interventions, and population awareness. The initiative emerged during a period of profound transformation for healthcare systems worldwide. Following the COVID-19 pandemic, the scientific community has increasingly recognized the need to reassess acute disease epidemiology while simultaneously addressing the organizational, educational, and technological factors that influence patient outcomes.

The articles published within this collection reflect this multidimensional perspective and demonstrate how the management of acute and time-dependent conditions extends well beyond the clinical encounter itself. The published studies highlight the importance of prevention, healthcare organization, workforce preparedness, epidemiological surveillance, and emerging technologies in improving population health.

Several contributions focused on the quality of care delivered to vulnerable populations. Bonacaro et al. [1] addressed the important issue of pain assessment in pediatric patients admitted to sub-intensive care units through a scoping review of the available evidence. Their work highlights persistent challenges in the recognition and measurement of pain among critically ill children and emphasizes the need for standardized assessment tools and evidence-based approaches to support clinical decision-making.

The impact of healthcare system organization was explored by De la Rosa et al. [2] through a comprehensive temporal analysis of emergency department visits in Northern Italy before and after the COVID-19 pandemic. Their findings contribute to a growing body of evidence demonstrating how major public health emergencies can profoundly alter healthcare utilization patterns and healthcare-seeking behaviors.

A broader epidemiological perspective was provided by Araujo et al. [3], who examined trends in congenital syphilis incidence and mortality in Brazil’s Southeast Region over a fourteen-year period. Their study highlights the importance of long-term epidemiological surveillance and public health monitoring in identifying persistent healthcare challenges and evaluating preventive interventions.

The organization and effectiveness of emergency response systems were further explored in the narrative review by Di Fronzo et al. [4], which examined the impact of advanced cardiovascular life support (ACLS) training on cardiac arrest management. The authors reinforce the importance of structured education programs in strengthening the chain of survival and improving emergency care preparedness.

The importance of prevention and health promotion emerged strongly in the study by Crotti et al. [5], who evaluated the feasibility of adapting Slovenia’s Health Promotion Centre model within the Province of Bergamo. Their findings provide valuable insights into the organizational factors that influence the successful implementation of population-based preventive interventions.

Technological innovation was represented by Raman et al. [6], who investigated the application of machine learning models for coronary heart disease prediction. By integrating SHAP-based interpretability approaches, the study demonstrates the growing potential of artificial intelligence to support risk stratification while maintaining transparency in clinical decision-making.

Innovations in emergency medicine are numerous and span multiple domains. These developments affect several areas, including education and training [7,8], as well as clinical practice and decision-making [9], both of which require continuous updating in light of the latest evidence and recent publications on cardiac arrest management [10]. Furthermore, research should continue to advance through the application of geospatial data analysis and the evaluation of emergency medical services (EMS) systems, thereby improving our understanding of healthcare delivery patterns and supporting more effective emergency care strategies [11,12].

Future research in acute diseases and emergency medicine should increasingly focus on strengthening the integration between emergency care systems and territorial healthcare services, ensuring a more seamless continuum of care from the community to the hospital. From this perspective, bridging prehospital emergency services, primary care, and community-based interventions will be essential to improving the early recognition, timely management, and appropriate triage of time-dependent conditions, meaning this could be relevant in various different settings [13,14]. The development of integrated care pathways supported by digital health tools, real-time data sharing, and interoperable information systems may further enhance coordination between hospital and territorial services. At the same time, emergency medical services (EMS) should be increasingly evaluated not only as isolated response systems but as key components of broader healthcare networks, where geospatial analysis, population health data, and community resources are combined to optimize responsiveness and equity of access. Strengthening this integration will be crucial to reducing fragmentation of care, improving outcomes in acute conditions, and ensuring that emergency medicine evolves in parallel with the needs of the populations it serves.

In conclusion, we would like to thank all contributors, researchers, clinicians, and educators whose ongoing efforts continue to advance the field of emergency medicine. Given the rapid evolution of evidence, technologies, and clinical practices, providing continuous updates and promoting lifelong learning are essential to ensuring the highest standards of patient care. Ongoing engagement with new research and emerging innovations remains crucial for improving outcomes and strengthening emergency healthcare systems.
